# Etiology analysis and G6PD deficiency for term infants with jaundice in Yangjiang of western Guangdong

**DOI:** 10.3389/fped.2023.1201940

**Published:** 2023-07-10

**Authors:** Yi-Kang Yang, Chun-Fan Lin, Fen Lin, Zi-Kai Chen, Yu-Wei Liao, Yu-Chan Huang, Bei-Ru Xiao, Shan-Hua Huang, Yu-Mei Xu, Yue-E. Chen, Yan-Bin Cao, Li-Ye Yang

**Affiliations:** ^1^ Institute of Medicine and Nursing, Hubei University of Medicine, Shiyan, China; ^2^ Precision Medical Lab Center, People’s Hospital of Yangjiang Affiliated to Guangdong Medical University, Yangjiang, China; ^3^ Department of Neonatology, People’s Hospital of Yangjiang Affiliated to Guangdong Medical University, Yangjiang, China; ^4^ Precision Medical Center, Chaozhou Central Hospital Affiliated to Southern Medical University, Chaozhou, China; ^5^ School of Life Science and Food Engineering, Hanshan Normal University, Chaozhou, China; ^6^ Yangjiang Branch, Biochip Beijing National Engineering Research Center, Yangjiang, China

**Keywords:** neonatal hyperbilirubinemia, neonatal jaundice, etiology, G6PD deficiency, hemolysis

## Abstract

**Objective:**

Glucose 6-phosphate dehydrogenase (G6PD) deficiency increases the risk of neonatal hyperbilirubinemia. The aim of this study is to evaluate the risk factors associated with hyperbilirubinemia in infants from the western part of Guangdong Province, and to assess the contribution of G6PD deficiency to neonatal jaundice.

**Methods:**

The term infants with neonatal hyperbilirubinemia in People's Hospital of Yangjiang from June 2018 to July 2022 were recruited for the retrospective analysis. All the infants underwent quantitative detection of the G6PD enzyme. The etiology was determined through laboratory tests and clinical manifestations.

**Results:**

Out of 1,119 term infants, 435 cases presented with jaundice. For the etiology analysis, infection was responsible for 16.09% (70/435), G6PD deficiency accounted for 9.66% (42/435), of which 3 were complicated with acute bilirubin encephalopathy), bleeding accounted for 8.05% (35/435), hemolytic diseases accounted for 3.45% (15/435), and breast milk jaundice accounted for 2.53% (11/435). One case (0.23%) was attributed to congenital hypothyroidism, multiple etiologies accounted for 22.3% (97/435), and 35.63% (155/435) were of unknown etiology. Of the jaundiced infants, 19.54% (85/435) had G6PD deficiency, while only 10.23% (70/684) of non-jaundiced infants had G6PD deficiency; this difference was found to be statistically significant (*P *< 0.001). Furthermore, the hemoglobin levels in the jaundiced infants with G6PD deficiency (146.85 ± 24.88 g/L) were lower than those without G6PD deficiency (156.30 ± 22.07 g/L) (*P* = 0.001). 65 jaundiced infants with G6PD deficiency underwent G6PD mutation testing, and six different genotypes were identified, including c.95A > G, c.392G > T, c.1024C > T, c.1311C > T, c.1376G > T, c.1388G > A, c.871G > A/c.1311C > T, c.392G > T/c.1388G > A, and c.1376G > T/c.1311C > T.65iciency

**Conclusion:**

In newborns in Yangjiang, G6PD deficiency, infection, and neonatal hemolytic disease were identified as the main causes of hyperbilirubinemia and acute bilirubin encephalopathy. Specifically, Hemolytic factors in infants with G6PD deficiency may lead to reduced hemoglobin and increased bilirubin levels in jaundiced infants.

## 1. Introduction

Neonatal jaundice, characterized by yellow skin, sclera and conjunctiva due to hyperbilirubinemia, is a common condition in Chinese newborns. Bilirubin can cross the blood-brain barrier, leading to severe hyperbilirubinemia, acute bilirubin encephalopathy, nuclear jaundice, and even permanent brain damage (1). In China, acute bilirubin encephalopathy in neonates is a significant concern, making it crucial to identify and intervene in risk factors for jaundice promptly. This approach can help reduce the incidence and severity of hyperbilirubinemia and acute bilirubin encephalopathy (2).

Neonatal hyperbilirubinemia (NHB) can arise from various causes, including infections as the primary cause, followed by perinatal and hemolytic factors (3, 4). Hereditary factors can also contribute to neonatal hyperbilirubinemia (5). This study aimed to comprehensively analyze the etiology of jaundiced infants in a hospital in the western part of Guangdong province, exploring the various factors causing this disease. Additionally, this study aimed to evaluate the contribution of glucose-6-phosphate dehydrogenase (G6PD) deficiency to neonatal jaundice.

## 2. Material and methods

### 2.1. Subjects

Full-term infants with a gestational age of >37 weeks, birth weight ≥2,500 g, and who underwent quantitative detection of G6PD enzyme were included in this study. The age of admission ranged from 1 to 28 days. Jaundiced neonates were identified as those whose maximum total serum bilirubin (TSB) reached or exceeded the 95th percentile of the hour-specific TSB nomogram established by the Chinese Multicenter Study Coordination Group for Neonatal Hyperbilirubinemia in 2015 (6). Demographic and clinical data of the participants were collected from their medical records. The diagnostic standard for severe hyperbilirubinemia was TSB ≥ 342 μmol/L (6).

This study obtained approval from the Ethics Committee of the People's Hospital of Yangjiang in 2022 (No.20220063). The patients' data was analyzed anonymously, and blood samples were used after clinical diagnosis; therefore, the Ethics Committee of the People's Hospital of Yangjiang granted a waiver for written consent.

### 2.2. Methods

General information was gathered, including the infant's sex, gestational age, birth weight, age and weight at admission, delivery mode, feeding pattern, as well as treatment measures received. Hemolytic disease of the newborn (ABO and Rh incompatibility), glucose-6-phosphate dehydrogenase (G6PD) deficiency, infections (sepsis, pneumonia, and urinary tract infections), extravascular hemorrhage (intracranial hematoma, scalp hematoma, gastrointestinal, and other bleedings), congenital hypothyroidism (diagnosed through neonatal screening), and breast milk jaundice were among the major clinical etiologies examined. Breast milk jaundice was diagnosed by exclusion, in infants who were exclusively breastfed and older than 14 days, with no other identifiable pathogenic factors for jaundice; As a result, breast milk jaundice was not considered as a contributing factor in the combined analysis. If a patient was found to have two or more contributing factors for jaundice, they were documented in both the combined group and the respective single factor groups. Information about acute bilirubin encephalopathy (typical clinical symptoms of the nervous system and/or MRI imaging) and criteria for blood exchange [as recommended by the American Academy of Pediatrics in 2022 (7)] was also documented.

ABO incompatibility hemolysis was tested by three serological antibody tests using commercial three-cell panel (LIBO biotechnology, China, Co, Ltd.) by gel technique. Three tests included red blood cells direct antiglobulin test (direct Coombs test), free antibody test (free) and antibody release test. Serological diagnostic criteria for ABO incompatibility hemolysis were as follows: (1) confirmed cases were neonates with two positive results of the three tests or with a positive result on the antibody elution test; (2) suspected cases were only positive for either direct Coombs test or serum free antibody test. Antibody elution test was the final confirmed diagnosis for neonatal hemolysis disease (8).

The confirmation of G6PD enzyme deficiency was made by measuring the production rate of NADPH, using a detection kit (Beijing Antu Bioengineering Co., Ltd., China) following the manufacturer's protocols. Infants with a production rate of NADPH lower than 2,500 U/L were categorized as G6PD-deficient (9).

To determine the frequency of G6PD deficiency in Yangjiang, routine body check-ups were conducted on male adults at our hospital, and they were also tested for G6PD deficiency using the same detection kit mentioned above. The deficiency was defined as G6PD enzyme activity lower than 1,300 U/L.

#### 2.2.1. Molecular diagnosis of G6PD deficiency

Blood specimens collected from G6PD-deficient patients were subjected to molecular analysis. For newborns with jaundice, whole blood was prospectively collected after clinical diagnosis and stored at −40°C in a biobank. DNA was extracted using a DNA extraction kit (HYBRIBIO Co, Ltd., China) according to the instructions and the quantity and purity were measured by NanoDrop One (Thermo Fisher Scientific Co., Ltd.). Amplification of G6PD gene was performed by PCR using VeritiTM Dx 96-Well Thermal Cycler (Thermo Fisher Scientific), followed by detection of the G6PD gene variant through reverse dot blot method (HYBRIBIO Co., Ltd., China) for 13 common G6PD mutation types, including c.95A > G (G6PD Gaohe), c.392G > T (G6PD Qing Yan), c.487G > A (G6PD Mahidol), c.493A > G (G6PD Taipei), c.592G > T (G6PD Coimbra), c.871G > A (G6PD Viangchan), c.1004C > T (G6PD Fushan), c.1024C > T (G6PD Chinese-5), c.1360C > T (G6PD Union), c.1376G > T (G6PD Canton), c.1387C > T (G6PD Keelung), c.1388G > A (G6PD Kaiping), *c.1381G > A* (G6PD Yunan) and *c.1311C > T* (polymorphism) (10).

### 2.3. Statistical analysis

The data was analyzed by SPSS 23.0 (IBM SPSS 23.0) and shown as mean ± standard deviation. Differences in continuous variables between the two groups were analyzed by the Mann–Whitney nonparametric test. The significance of differences in the categorical variables was determined by the Chi-square test or Fisher's exact test. *P* < 0.05 was considered statistically significant.

## 3. Results

### 3.1. Demographic and clinical characteristics

**Figure 1:**
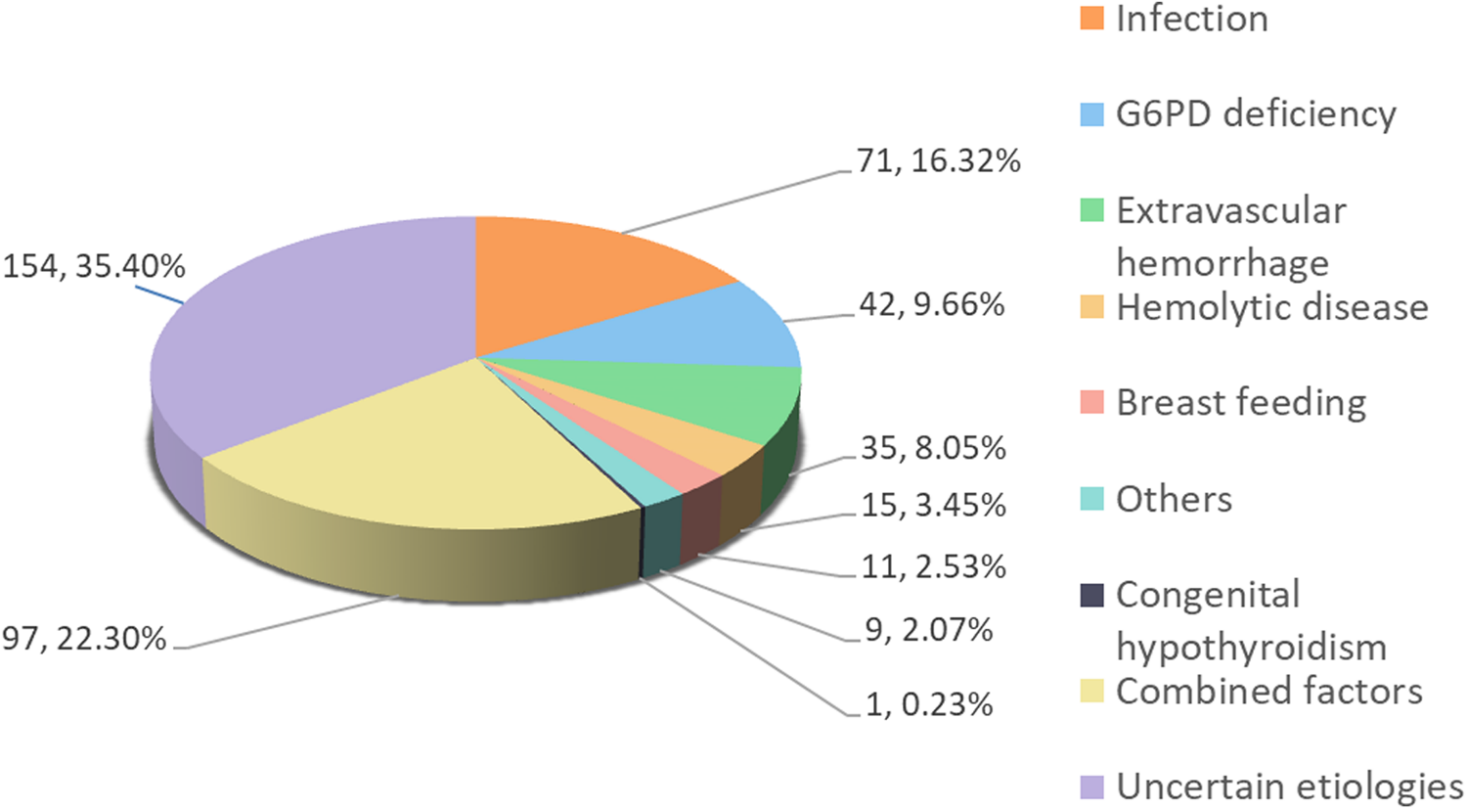
Clinical causes in 435 cases of jaundice. Combined factors, with 2 or more clinical causes.

From June 2018 to July 2022, a quantitative assay of G6PD enzyme was conducted on 1,828 in-hospital infants. Of these, 1,119 were term infants, with 435 presenting with jaundice and 684 presenting normal bilirubin levels. G6PD deficiency accounted for 9.66% (42/435) hyperbilirubinemia cases, while multiple etiologies, including infection (pneumonia, sepsis, and upper respiratory tract infection), hemorrhage (delivery injury, scalp hematoma, gastrointestinal bleeding), ABO incompatibility hemolysis, and breast-milk jaundice were responsible for 22.3% (97/435), 16.09% (70/435), 8.05% (35/435), 3.45% (15/435), and 2.53% (11/435) of cases, respectively. One case (0.23%, 1/435) was attributed to congenital hypothyroidism, and 2.07% (9/435) of cases were caused by other factors such as thalassemia, mothers with diabetes, and hereditary spherocytosis. The etiology was unknown in 35.63% (155/435) of cases (Figure 1).

Among the 435 cases of jaundice, severe hyperbilirubinemia (TSB ≥342 μmol/L) was observed in 50 cases, 8 cases of which presented with acute bilirubin encephalopathy (2 males and 6 females), and 7 cases required exchange transfusions. The primary causes of acute bilirubin encephalopathy were G6PD deficiency (3 cases) and hemorrhage (3 cases), followed by infection (2 cases of sepsis and 1 case of pneumonia), ABO incompatibility hemolysis (2 cases), and congenital hypothyroidism (1 case) (Table 1). One female infant with acute bilirubin encephalopathy was admitted at the age of 17 days, who had congenital hypothyroidism combined with G6PD deficiency and had not undergone neonatal screening at birth (Table 1).

**Table 1:** Demographic and clinical characteristics of 8 infants with acute bilirubin encephalopathy.

S/N	Diagnosis	Sex	Age (day)	Gestational age (weeks)	Birth weight (g)	Delivery mode	Feeding method	G6PD (U/L)^a^	TBIL (μmol/L)	DBIL (μmol/L)
1	1. Acute bilirubin encephalopathy 2. Neonatal hyperbilirubinemia 3. Neonatal ABO hemolytic jaundice 4. Anemia	M	5	39 + 2	2,700	Cesarean	Breast feeding	3,457	493.5	19.62
2	1. Acute bilirubin encephalopathy 2. Neonatal hyperbilirubinemia 3. Neonatal sepsis 4. Coagulopathy 5. Gastrointestinal bleeding	F	10	38 + 6	2,700	Vaginal	Breast feeding	3,425	401.7	40.73
3	1. Acute bilirubin encephalopathy 2. Neonatal sepsis 3. Neonatal hyperbilirubinemia 4. Anemia 5. Metabolic acidosis 6. Neonatal hyperglycemia 7. Gastrointestinal bleeding 8. Myocardial damage	F	8	38 + 3	2,900	Vaginal	Mixed feeding	2,786	597	52.3
4	1. Acute bilirubin encephalopathy 2. Neonatal hyperbilirubinemia 3. G6PD deficiency 4. Anemia	M	6	38 + 1	3,000	Vaginal	Breast feeding	774	634.9	46.47
5	1. Acute bilirubin encephalopathy 2. Neonatal hyperbilirubinemia 3. Neonatal ABO hemolytic disease 4. Cerebral hemorrhage 5. Myocardial damage 6. Mild anemia	M	6	39 + 1	2,700	Cesarean	Breast feeding	3,457	500.9	44.58
6	1. Acute bilirubin encephalopathy 2. Neonatal hyperbilirubinemia 3. Congenital hypothyroidism 4. G6PD deficiency 5. Anemia	F	17	41 + 4	3,300	Vaginal	Breast feeding	695	471.54	14.88
7	1. Acute bilirubin encephalopathy 2. Neonatal hyperbilirubinemia 3. Anemia 4. Conjunctival hemorrhage 5. Fundus hemorrhage	F	7	38 + 3	3,500	Vaginal	Breast feeding	5,501	555.6	19.6
8	1. Acute bilirubin encephalopathy 2. Neonatal hyperbilirubinemia 3. Anemia 4. Pneumonia 5. G6PD deficiency	F	7	38	2,650	Cesarean	Mixed feeding	960	499.4	12.48

M, male; F, female.

^a^ G6PD enzyme of <2,500 U/L was defined as G6PD deficiency.

### 3.2. G6PD deficiency prevalence

Of the 435 cases with jaundice, 19.54% (60 males, 25 females) were found to be G6PD-deficient, while only 10.23% (47 males, 23 females) of the 684 cases with normal bilirubin levels were G6PD-deficient. The prevalence of G6PD deficiency was significantly higher in infants with jaundice compared to their counterparts (*P* < 0.001) (Table 2). The prevalence of G6PD-deficient in severe hyperbilirubinemia (TSB ≥ 342 µmol/L) and mild-medium jaundice (TSB < 342 µmol/L) was 30% (15 out of 50, 11 males, 4 females) and 18.18% (70 out of 385, 49 males, 11 females), respectively. A statistically significant difference was observed between the two groups (*P* = 0.041) (Table 3). In comparison, G6PD deficiency was present in 8.75% (42/480) of male adults during routine body check-ups.

**Table 2:** The comparison of G6PD deficiency in the infants with jaundice and group with normal bilirubin levels.

	Hyperbilirubinemia (*n*)	Normal bilirubin (*n*)	Total (*n*)	*P* value
G6PD deficiency	85	70	155	<0.001
Normal G6PD	350	614	964	
Total	435	684	1,119	

**Table 3:** G6PD deficiency in the infants with severe and mild-medium jaundice.

G6PD state	Severe hyperbilirubinemia	Mild-medium hyperbilirubinemia	Total	*P* value
G6PD deficiency	15	70	85	0.041
Normal G6PD	35	315	350	
Total	50	385	435	

### 3.3. Comparison of bilirubin and hemoglobin levels in infants with jaundice

Of the 435 infants with jaundice, one case was excluded due to the lack of hemoglobin data. The remaining cases were grouped according to jaundice causes—normal, G6PD-deficient, ABO incompatibility hemolysis, and ABO incompatibility hemolysis combined with G6PD-deficiency. The difference in total bilirubin levels among these four groups was compared (Table 4), and the peak bilirubin values were not statistically significant (*P* = 0.95). Among infants with jaundice, the hemoglobin levels of 80 G6PD-deficient infants were 146.85 ± 24.88 g/L, which was significantly lower than that of infants with normal G6PD (156.30 ± 22.07 g/L) (*P* = 0.001) (Table 5). Meanwhile, the hemoglobin levels of 27 infants with ABO incompatibility hemolysis were at 134.33 ± 24.18 g/L, slightly lower than that of infants with G6PD deficiency (146.85 ± 24.88 g/L) (*P* = 0.014).

**Table 4:** The comparison of peak bilirubin levels in infants with hyperbilirubinemia.

Group	Case (*n*)	TBIL (μmol/L)^a^	*H* value	*P* value
Normal G6PD	322	278.65 (222.88–308.10)	0.35	0.95
G6PD deficiency	80	278.25 (216.93–308.58)		
ABO hemolysis	27	253 (196.90–339.20)		
ABO hemolysis/G6PD deficiency	5	267.8 (220.70–321.00)		

TBIL, total bilirubin.

^a^ Median (95% confidence interval).

**Table 5:** Comparison of hemoglobin levels in infants with jaundice.

Group	Case (*n*)	HB (g/L)^a^	95% CI	*F* value	*P* value
Normal G6PD	322	156.30 ± 22.07	153.88–158.72	11.48	<0.001
G6PD deficiency	80	146.85 ± 24.88	141.31–152.39		
ABO hemolysis	27	134.33 ± 24.18	124.77–143.90		
ABO hemolysis/G6PD deficiency	5	133.40 ± 23.58	102.12–164.68		

^a^ Mean ± standard deviation.

### 3.4. G6PD genotypes distribution

A total of 65 blood samples from jaundiced infants with G6PD deficiency were analyzed for G6PD genotypes using reverse dot hybridization (10). Five types of gene mutations and one polymorphism were detected in infants with jaundice, namely c.95A > G, c.392G > T, c.1024C > T, c.1376G > T, c.1388G > A, and c.1311C > T (polymorphism). Furthermore, three kinds of compound heterozygous mutations were identified, namely c.871G > A/c.1311C > T, c.392G > T/c.1388G > A, and c.1376G > T/c.1311C > T (Table 6). Specifically, c.871G > A was consistently linked to 1311C > T in all nine cases (Table 6). Moreover, one G6PD-deficient infant developed acute bilirubin encephalopathy with compound heterozygous mutations of c.392G > T and c.1388G > A.

**Table 6:** G6PD genotypes distribution in 65 cases of G6PD deficient infants with hyperbilirubinemia.

Mutation	Hemizygote (*n*)	Heterozygote (*n*)	Homozygote (*n*)	Total (*n*)	Percentage %
Wild type	1	1	0	2	3.1
c.95A > G	5	0	0	5	7.7
c.392G > T	3	0	0	3	4.6
c.1024C > T	2	1	0	3	4.6
c.1311C > T	1	0	0	1	1.5
c.1376G > T	10	3	1	14	21.5
c.1388G > A	21	3	0	24	36.9
c.871G > A/c.1311C > T	6	3	0	9	13.8
c.392G > T/c.1388G > A	0	1	0	1	1.5
c.1376G > T/c.1311C > T	0	3	0	3	4.6
Total	49	15	1	65	100

## 4. Discussion

The pathogenic factors of neonatal hyperbilirubinemia are multifaceted, and different cases can have a single or mixed etiology. Common causes include extravascular hemorrhage, hemolytic diseases, infection, G6PD deficiency, breastfeeding, and maternal disease factors. Furthermore, the etiological composition of neonatal hyperbilirubinemia can vary in different regions. Among the 435 full-term infants with hyperbilirubinemia in our study, the main causes were combined factors (22.3%), infection (16.32%), G6PD deficiency (9.66%), hemorrhage (8.05%), hemolytic diseases (3.45%), breast milk jaundice (2.53%), other factors (2.07%), and congenital hypothyroidism (0.23%).

A study conducted in the eastern region of China reported that the top five pathogenic factors of neonatal unconjugated hyperbilirubinemia were combined factors (16%), infections (15%), breast milk jaundice (11%), hemorrhage (10%), hemolytic diseases (7%), and G6PD deficiency (1%) (3). In comparison, the cohort in our study had a higher prevalence of G6PD deficiency, likely due to the high prevalence of G6PD deficiency in the population of western Guangdong province. Nevertheless, infections, hemolytic diseases, and hemorrhage remained the main pathogenic factors of neonatal jaundice in our study.

In this study, the prevalence of G6PD deficiency was 19.54% in the jaundiced group, while it was 10.23% in the control group with normal bilirubin levels. In Chaozhou of eastern Guangdong province, among 882 neonates with hyperbilirubinemia, 74 cases (8.39%) were G6PD-deficient (11), and in Fujian province, the prevalence of G6PD deficiency among neonates with hyperbilirubinemia was 7% (12). As such, the prevalence of G6PD deficiency among neonates with hyperbilirubinemia in the western part of Guangdong is higher compared to that in eastern Guangdong and Fujian province.

Multiple reports have demonstrated that G6PD-deficient infants have a significantly higher predisposition to neonatal jaundice and are more susceptible to acute bilirubin encephalopathy (2, 4, 7). In our study, the prevalence of G6PD deficiency was 30% in infants with severe jaundice and 18.18% (70/385) in infants with mild-medium jaundice. Of the neonates with acute bilirubin encephalopathy, 37.5% (3 in 8) were G6PD-deficient. Moreover, the frequency of G6PD deficiency in Yangjiang males was 8.75%, which was higher than that of the entire Guangdong province (13). In the voluntary Kernicterus Registry in the United States, 20.8% of 125 affected newborns were G6PD-deficient, while the male frequency of G6PD deficiency was estimated to be 0.5%–2.9% (14). Our study, along with previous research, indicates that G6PD-deficient infants are predisposed to neonatal jaundice, and even to kernicterus.

In 2004, the American Academy of Pediatrics (AAP) suggested that G6PD deficiency should be considered as a high-risk factor for jaundice in newborns ≥35 weeks old, and should therefore be evaluated in the diagnosis and treatment of jaundiced newborns (15). In clinical practice, given the relatively high prevalence of G6PD deficiency in this population, G6PD screening for all local newborns is required. Furthermore, transcutaneous bilirubin monitoring is strongly recommended when an infant with G6PD deficiency is discharged. Early detection is beneficial for prompt treatment, which is in accordance with the AAP guidelines. These improvements in neonatal care could decrease neonatal morbidity and mortality in this region.

G6PD deficiency is the most prevalent inherited enzyme deficiency disease, yet the mechanism of neonatal hyperbilirubinemia resulting from G6PD deficiency remains incompletely understood (4). Previously, it was believed that jaundice in infants with G6PD deficiency was mainly caused by excessive bilirubin production during hemolysis (16–18). Nevertheless, some studies have found minimal evidence of hemolysis in jaundiced neonates with G6PD deficiency (19–21). In our cohort of infants with hyperbilirubinemia, the hemoglobin levels in the G6PD-deficient group were significantly lower than those in the normal G6PD group (*P* < 0.001), and slightly higher than those in the ABO hemolysis group (134.33 ± 24.18 g/L) (*P* = 0.014). Since fetal erythropoiesis in infants with G6PD deficiency was the same as that in controls, and G6PD was dispensable for human erythroid cell differentiation (22, 23). Our findings suggest that decreased hemoglobin levels may be due to hemolytic factors in jaundiced infants with G6PD deficiency. Another possible explanation is the disruption of the oxidant-antioxidant balance and impaired recycling of peroxiredoxin 2, which can impact bilirubin clearance (4). Moreover, co-inheritance of a uridine diphosphate glucuronosyltransferase 1A1 (UGT1A1) gene variant is an additional risk factor for neonatal jaundice in G6PD-deficient infants (11).

G6PD is caused by loss-of-function mutations in the G6PD gene and follows an X-linked recessive inheritance pattern. The distribution of G6PD deficiency is predominantly found in the south of the Yangtze River in China, with Guangdong province exhibiting a high incidence of G6PD deficiency (13). Interestingly, common variants among G6PD-deficient individuals in southern China are unique to these populations (13, 24). In our study, Canton (c.1376 G > T) and Kaiping (c.1388 G > A) were the most frequent variants, accounting for over 78% of G6PD-deficient infants with jaundice. This distribution pattern was consistent throughout Guangdong province and the entire country (13, 25).

In summary, neonatal hyperbilirubinemia and acute bilirubin encephalopathy in Yangjiang were primarily caused by G6PD deficiency, infections, and hemolytic disease of the newborn. Compared to other parts of China, the high prevalence of G6PD deficiency in the western region of Guangdong made it the predominant factor for neonatal hyperbilirubinemia. In our cohort of infants with jaundice, the G6PD-deficient group had significantly lower hemoglobin levels than the normal G6PD group, suggesting that hemolytic factors in this group may contribute to reduced hemoglobin and increased bilirubin levels in jaundiced infants. We strongly recommend G6PD screening and transcutaneous bilirubin monitoring for all newborns in this area to improve neonatal healthcare.

## Data Availability

The raw data supporting the conclusions of this article will be made available by the authors, without undue reservation.
